# American Medical Extended Reality Association Expert Consensus: Best Practices for Patient and Public Involvement in Medical Extended Reality Research

**DOI:** 10.1177/29941520251377071

**Published:** 2025-09-18

**Authors:** Tom Norris, Ella Tetrault, Muskaan Mehra, Karisma Suchak, Lindsey Ross, Jan Ballesteros, Brennan M.R. Spiegel

**Affiliations:** ^1^ American Chronic Pain Association (ACPA), Los Angeles, CA, USA.; ^2^ World Patients Alliance (WPA), Los Angeles, CA, USA.; ^3^ U.S. Pain Foundation, Los Angeles, CA, USA.; ^4^ Division of Health Services Research, Department of Medicine, Cedars-Sinai Medical Center, Los Angeles, CA, USA.; ^5^ Department of Neurosurgery, Cedars-Sinai Medical Center, Los Angeles, CA, USA.; ^6^ Providence Saint John’s Health Center, Orthopaedic & Spine Center, Santa Monica, CA, USA.; ^7^ Department of Medicine, Cedars-Sinai Medical Center, Los Angeles, CA, USA.

**Keywords:** patient and public involvement, patient engagement, patients with lived experiences, medical extended reality, patient, caregiver, co-design, co-production, Patient-Centered Outcomes Research Institute, National Institutes of Health, Congressionally Directed Medical Research Program

## Abstract

Patient and public involvement (PPI) is critical in medical extended reality (MedXR) research to ensure that interventions are relevant, patient-centered, and impactful. This expert consensus from the American Medical Extended Reality Association offers a practical framework for integrating patient voices throughout the research lifecycle, from study design to post-study feedback. Drawing on principles from the Patient-Centered Outcomes Research Institute, Congressionally Directed Medical Research Programs, and the National Institutes of Health, it highlights strategies for meaningful involvement of patient partners, emphasizing inclusivity, reciprocity, and transparency. Key areas of focus include best practices for recruiting and compensating patient partners, ensuring diversity and representation, simplifying communication, and addressing ethical considerations. The expert consensus addresses challenges in patient engagement specific to MedXR, offering solutions such as iterative feedback loops during prototype development and collaborative safety monitoring. Case studies provide real-world examples of how PPI insights have enhanced MedXR research, improving accessibility, relevance, and equity. This document serves as a comprehensive resource for researchers, developers, and clinicians, supporting the development of MedXR interventions that are not only scientifically rigorous but also deeply aligned with patient and public needs and priorities.

## Introduction

The field of medical extended reality (MedXR), encompassing uses of virtual reality (VR), augmented reality (AR), and mixed reality (MR), is transforming health care by enhancing research and patient care through immersive technologies.^1^ As MedXR moves from experimental stages to patient-facing applications,^2–7^ integrating patient perspectives has become essential. Institutions like the National Institutes of Health (NIH), Congressionally Directed Medical Research Programs (CDMRP), and the Patient-Centered Outcomes Research Institute (PCORI) emphasize, and often require, documented patient and public involvement (PPI) for clinical trial approval and funding.^8,9^

Despite these initiatives, there remains limited specific guidance on how patients with lived experiences (PWLE) can be systematically integrated into MedXR research.^10–12^ Given MedXR’s interactive and experimental nature, patient insights are crucial from initial design through implementation and evaluation. PPI is a continuous process.^10,13–15^ Effective PPI helps shape study design and research questions, create accessible materials, including consent documents, support participant retention, and ensure protocols reflect real-world patient needs, serving as a crucial bridge between scientific research and patients’ everyday experiences.^16–21^ Engaged patients further help develop recruitment and retention strategies and foster trust and transparency between researchers and participants, grounded in lived experiences.^13,22,23^

This American Medical Extended Reality Association (AMXRA) expert consensus, led by a patient advocate experienced in both MedXR research and XR for chronic pain management and researchers in the field, offers best practices for PPI in MedXR research. This framework recognizes the contributions of all stakeholders while promoting MedXR interventions that are scientifically robust and patient-centered. It prioritizes approaches that ensure interventions are effective, equitable, and compassionate in patient care.

## Principles of Patient and Public Involvement

PPI encompasses a wide range of voices—including PWLE, caregivers, people with disabilities, and those from underserved communities. Their involvement leads to MedXR tools that are scientifically robust and meaningful to end users.^10,24,25^ Drawing from frameworks by PCORI, NIH, and CDMRP that stress the value of involving patients as active partners across the research process, this section outlines core PPI principles tailored to MedXR’s unique demands.^8,22,26–30^

### Meaningful involvement across all research phases

Effective PPI ensures that patients, caregivers, clinicians, and other stakeholders are integral to every stage, from planning through post-study feedback.^22^ In MedXR, co-defining study objectives ensures the work remains grounded in real-world needs, enhancing the relevance and adoption of the resulting tools.^30^

### Reciprocity and co-learning

PCORI highlights reciprocity and co-learning as core to meaningful PPI, promoting mutual respect and knowledge exchange between researchers and patients.^30^ This collaborative learning enriches the research: patients gain insights into the research process, and researchers deepen their understanding of lived experiences. MedXR research especially benefits from this, as patients provide feedback on usability while gaining insight into the research process. Close collaboration ensures MedXR tools are not only technically advanced but also user-friendly and accessible for patients of various backgrounds and medical histories.^16,25,30–32^

### Fostering trust, transparency, and honest communication

Trust is essential in MedXR research, where unfamiliar technologies can feel intimidating to patients.^22^ Both NIH and PCORI emphasize the importance of open, honest communication among researchers, patients, and other stakeholders.^22,31^ Providing clear explanations of research goals, methods, and risks, which include potential adverse events (AEs), and expected outcomes, helps build confidence and align expectations. Acknowledging study limitations and uncertainties fosters respectful, two-way dialogue.^13^

Transparency also encourages patients to share important medical information, reinforcing the ethical principles of confidentiality and justice.^33^ By safeguarding patient privacy and promoting fairness, researchers create a secure and respectful environment for participation.^13^

Including patients on ethical review boards strengthens both accountability and trust, especially when working with vulnerable populations.^30,31^ When patients feel heard and valued, they are more likely to engage actively.^34^ Open discussions about the purpose, risks, and potential benefits of MedXR interventions demonstrate a genuine commitment to patient-centered care. This trust-centered approach not only enhances collaboration but also ensures that research is grounded in the real-world needs of those it aims to serve.

### Relevance to patient needs

PPI ensures research aligns with patient experiences and improves the usability and adoption of MedXR tools. NIH emphasizes involving patients in informed consent development to ensure clarity and relevance for informed decision-making.^35,36^ A later case study in this document shows how PPI enhanced the accessibility and transparency of study materials for an NIH-funded MedXR trial.

When end users are not involved early, valuable resources are often wasted, and promising innovations go unused, particularly in health care.^37,38^ Even well-designed tools can fail in real-world settings due to high costs, poor user experience, lack of validation, unintended side effects, limited trust, or cultural mismatches—challenges that are especially critical in fast-evolving fields like MedXR.^39,40^ To avoid these pitfalls, integrating diverse stakeholder perspectives, particularly those of end users such as patients and frontline staff, who are often overlooked in traditional approaches, is essential. Their early involvement helps guide more informed decisions about resource allocation, supporting the successful adoption, implementation, and long-term sustainability of digital health interventions like MedXR across varied health care settings.^40^

Despite their potential, mHealth innovations struggle with adoption without meaningful input from end users.^41–43^ Furthermore, a 2021 Implementation Science review identified limited stakeholder engagement as a key reason why promising interventions often fail to scale.^38^ Engaging patients early and meaningfully in the development process not only mitigates risk but also enhances the likelihood of real-world impact, ultimately creating greater value for developers, funders, and researchers.^38^

### Meaningful patient partnership

While engaging patients in all phases of MedXR research is crucial, it is equally important to avoid tokenistic involvement.^39^ Meaningful PPI means ensuring that patient input is not only sought but also genuinely considered and integrated into research decisions. This requires clear communication channels, transparent decision-making processes, and a willingness to adapt research plans based on patient feedback. Researchers should proactively seek diverse perspectives and ensure that patients feel empowered to share their insights and challenge existing assumptions.^40^ Ultimately, meaningful PPI aims to shift power dynamics, creating a truly collaborative research environment where patients and researchers work together as equal partners.^10,44–47^

### Accessibility and distribution of research

To advance patient-centered research and ensure broad accessibility for researchers and health professionals, this expert consensus was submitted to an open-access journal and will be presented at public-facing workshops. Additionally, to enhance the reach, visibility, and comprehensibility of the findings, clear, accessible summaries, infographics, and press releases can be created. Collaborations with hospitals, research institutions, and social media platforms will increase public engagement. Community discussions, open forums, and patient advocate networks will help disseminate information to those who may benefit the most. These efforts aim to maximize the impact of research on clinical practice and public understanding.^46,48^

### Continuous feedback and collaboration

PPI is not a one-time event, but an ongoing process.^14^ NIH, PCORI, and CDMRP all advocate for continuous feedback and collaboration throughout the research study. This means regularly checking in with patients, gathering their input, and adjusting the study protocol as needed to reduce patient burden or improve the relevance and effectiveness of the research.

In MedXR research, continuous feedback is essential, given the iterative nature of technology development.^30,31,49–52^ As prototypes evolve through cycles of testing and refinement, input from patients and the public plays a pivotal role in shaping interventions that are both effective and user-centered. Sustained collaboration throughout this process helps ensure that the final product aligns closely with patient needs, preferences, and expectations.

Through continuous feedback and collaboration, we also uphold both ethical tenets of beneficence and non-maleficence.^53^ Beneficence is an ethical value that we do not cause harm to those whom we are trying to help, and non-maleficence is the obligation not to harm patients. Through feedback and collaboration, we learn from the patients if such treatment interventions result in new unknown or unanticipated problems and AEs.

### Ethical and practical considerations

Engaged patients play a key role in the ethical oversight of research to ensure that the process is aligned with patient values and ethical standards.^53^ NIH emphasizes that PPI contributes to ensuring that clinical trials respect patients’ rights, needs, and preferences. Patients can provide valuable input on ethical issues that arise from research processes, such as the design of informed consent documents and the handling of sensitive data.

In MedXR research, where new technologies may raise novel ethical concerns, such as data privacy issues and the potential psychological impact on users, PPI is important.^49,54–56^ By involving patients in ethical discussions, researchers can navigate these challenges more effectively and ensure that the research adheres to the highest standards of patient participant autonomy, care, and respect.

### Training support for clinicians and researchers

In addition to involving patient partners in the research process, it is paramount to also equip researchers with the skills to incorporate patient insights into their studies to enhance the relevance and feasibility of studies.^13,32,57,58^ Continuous professional development opportunities, including workshops and peer learning sessions, can also help clinicians and researchers stay updated on best practices in patient engagement as well as enhance active listening. By providing the necessary educational and awareness tools, resources, and ongoing support, organizations can empower researchers and clinicians to engage patients meaningfully, ultimately leading to more patient-centered research.

### Identifying, recruiting, and compensating patient partners

Finding the right patient advisors is a crucial step in ensuring that MedXR research is genuinely patient-centered. Ideal advisors bring lived experiences and insights into the condition under study, enhancing the research with authentic, real-world perspectives, even without technical expertise in MedXR.

Researchers can identify potential partners through various avenues. Beyond connecting with patients from previous clinical trials who have demonstrated commitment to research, advocacy groups, community organizations, and health systems with existing patient advisory councils offer valuable resources. These groups help identify individuals who not only understand the challenges of their condition but also recognize the importance of advancing research. Collaborating with community-based organizations, public health agencies, senior centers, housing programs, and transportation services can further support recruitment efforts. Partnerships with both nonprofit and for-profit patient advocacy groups also broaden the pool of potential advisors. Additionally, social marketing strategies—including mass media, social media, and public spaces like religious institutions, libraries, and schools—can help raise awareness and attract patient partners.^59^

To ensure inclusive participation, research teams should address common barriers by providing practical support, such as transportation, meals, and technology access, while also recognizing and thanking patient partners for their contributions. These individuals should encompass a diverse range of socioeconomic backgrounds and health experiences to ensure the trial accurately reflects the needs and priorities of the broader patient population. In the context of MedXR, it is essential to include varying levels of digital and eHealth literacy to inform the design of systems, enhance health outcomes, and promote health equity in the digital age.^60–63^

Effective recruitment of both PWLE partners and participants requires transparent communication about roles, expectations, and the impact of their contributions.^19,64^ Fair compensation, which includes covering time, expenses, and expertise, acknowledges their vital role and should be budgeted as a line item, often through stipends or honorariums.^47,64^

By carefully identifying, recruiting, and compensating patient partners, researchers can cultivate strong, collaborative relationships that center on patient needs, fostering trust, transparency, and relevance in MedXR research.^64,65^

## Stages of MedXR Research and PPI

**FIG. 1.**
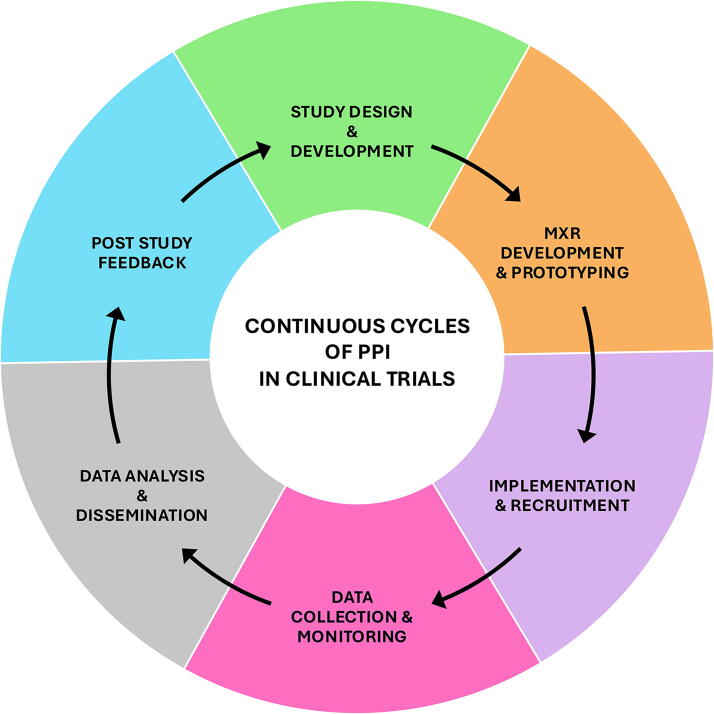
Patient engagement across the research lifecycle matrix. This figure illustrates the continuous cycle of PPI throughout six key phases of a clinical trial: Study Design & Development, MedXR Development & Prototyping, Implementation & Recruitment, Data Collection & Monitoring, Data Analysis & Dissemination, and Post-Study Feedback. It emphasizes how PPI is integrated at each stage, reinforcing their ongoing role in the research process. PPI, patient and public involvement.

**FIG. 2.**
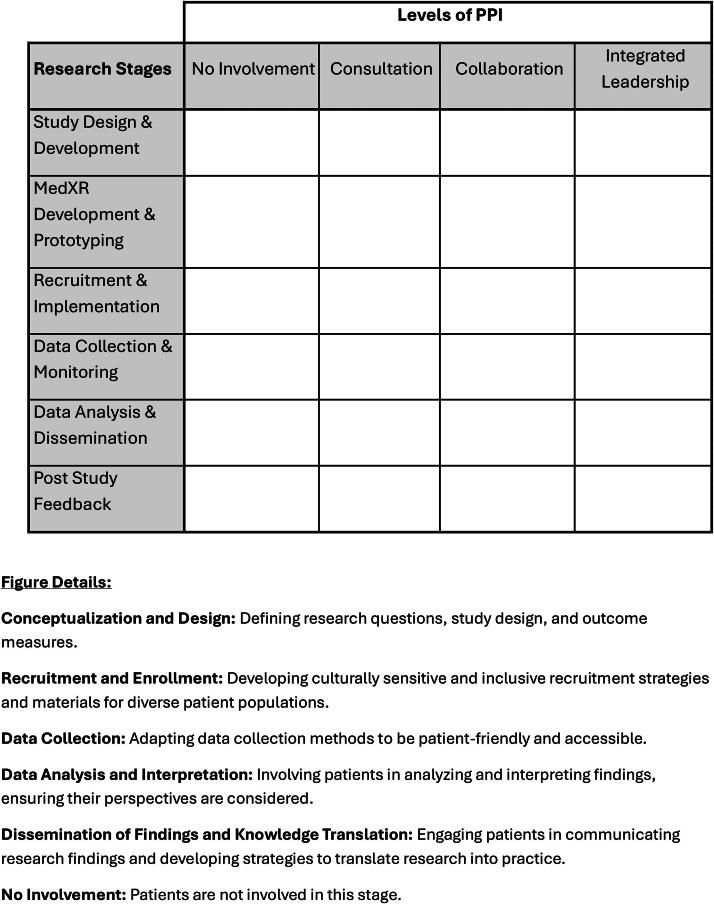
Mapping levels of PPI across research stages. This matrix enables researchers to systematically track the extent of PPI across the research process and identify areas for enhancing engagement. Definitions for the rows and columns are provided below. PPI, patient and public involvement.

**FIG. 3.**
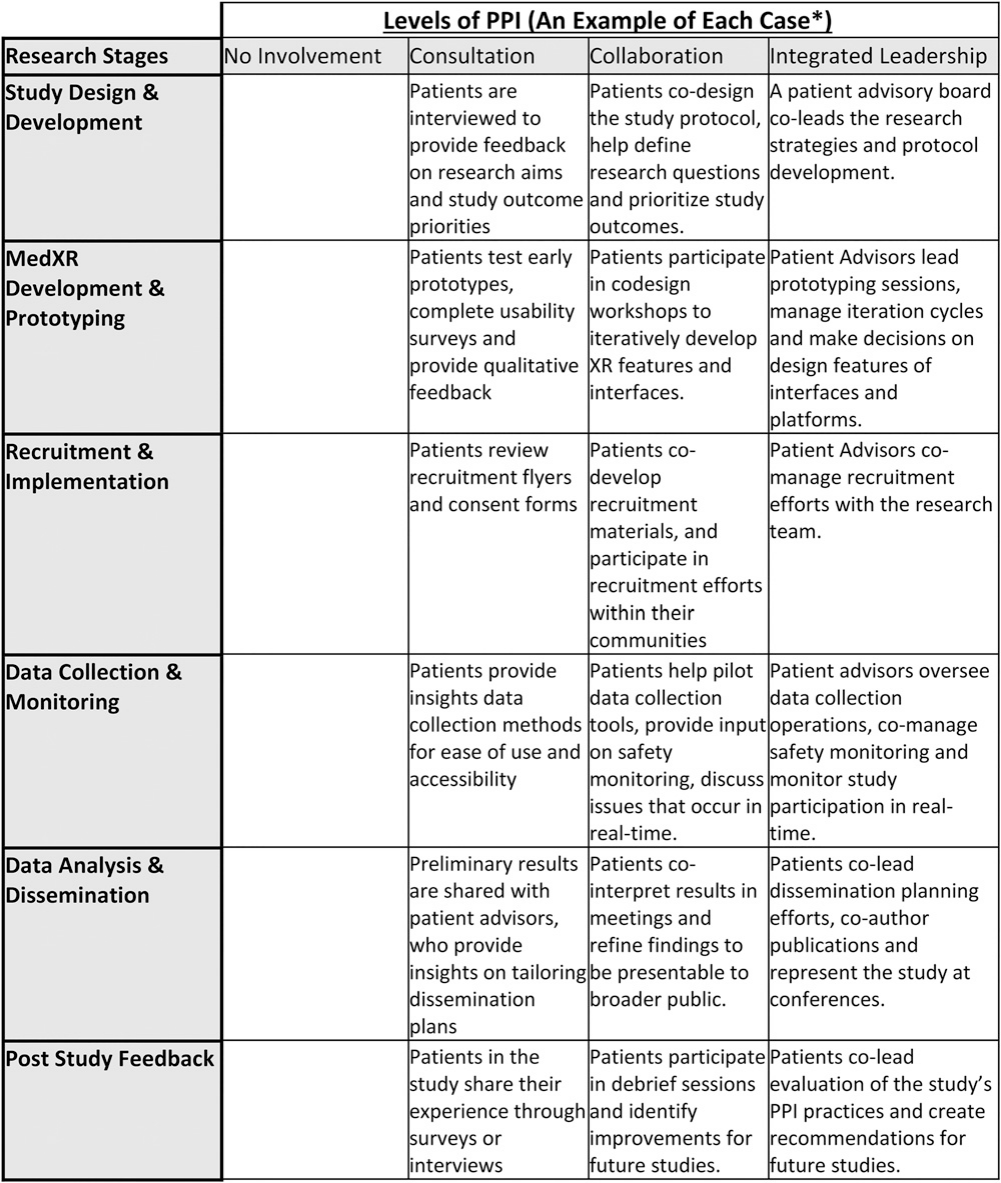
Practical application of mapping levels of PPI across research stages. This matrix exemplifies how the different levels of PPI involvement across the research stages can be envisioned in general. *These examples are limited to one example per column and row, but PPI can occur in multiple ways for each stage. PPI, patient and public involvement.

**FIG. 4.**
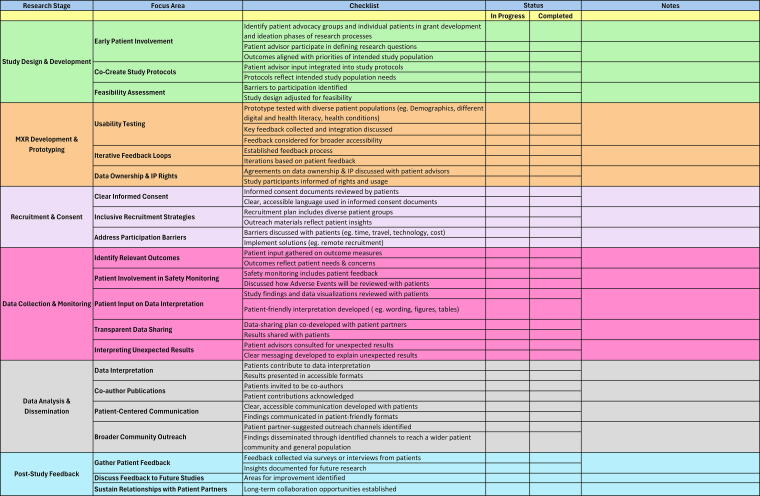
Patient engagement checklist: requirements across areas of research. This figure enables comprehensive tracking of patient involvement across multiple research stages, outlining specific requirements for each and offering a detailed checklist to monitor progress and evaluate the status of *key* items at every stage of the research process: (1) Study Design & Development; (2) MedXR Development & Prototyping; (3) Recruitment & Implementation; (4) Data Collection & Monitoring; (5) Data Analysis & Dissemination; (6) Post-Study Feedback.

**FIG. 5.**
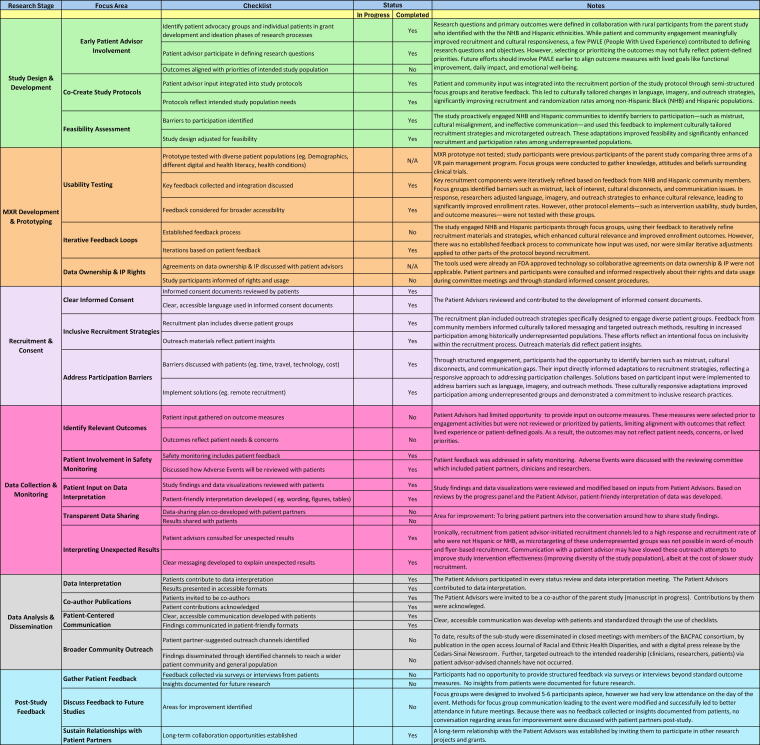
Practical application of the checklist. This figure provides a detailed example of how to apply the checklist from Fig. 4, using Case Study #1. The status and notes sections reflect whether each patient engagement activity was implemented, deemed not applicable, or unaddressed. While not a perfect example, this case offers a practical overview of how to use the checklist and highlights both the strengths and areas where patient engagement could be improved in future iterations of the study.

PPI should span all stages of the MedXR research lifecycle, ensuring interventions are truly patient-centered and impactful. Engaging patients from the outset and maintaining their involvement strengthens the research. Figure 1 presents an overview of PPI across the research lifecycle matrix, while Figure 2 maps the levels of PPI across research stages to help researchers track and enhance patient involvement while identifying areas for improvement. Figure 3 provides examples of various levels of PPI throughout research areas—from consultative to collaborative to integrated leadership roles. Finally, Figure 4 presents a checklist outlining the key requirements for patient engagement at each stage of research, and Figure 5 illustrates its practical application through a case example, specifically Case Study #1.

### Study design and development

#### Study ideation and scientific research plan development

Engage patient partners at the planning stage to help define research questions and relevant outcomes, ensuring the study is grounded in patient priorities and real-world needs. Patient insights can guide the creation of meaningful, patient-centered interventions rather than products that lack a clear use case.^19,32,66^

#### Co-creation of study protocols

Collaborate with patients to design protocols, including data collection methods and outcome measures. Patient input ensures that the study’s approach resonates with the patient population, leading to more relevant data. While no technical expertise is required, patients’ lived experiences offer invaluable insights that researchers can translate into scientifically sound protocols.

#### Feasibility assessment

Collaborate with patients to assess the study’s feasibility from their perspective, addressing potential barriers like participation burden and accessibility. Patient feedback can help streamline study elements, such as reducing unnecessary measurements, enhancing the practicality of the design, and improving recruitment and retention. This is especially important in MedXR research, where unfamiliar technologies can steepen the learning curve. Developing clear instructions and streamlined questionnaires is essential to avoid overwhelming participants.

### MedXR development and prototyping

While literature on developing XR technologies in health care with patient partners is still limited, codesign principles from broader mHealth interventions offer a valuable foundation for developing effective MedXR technologies. As outlined by Eyles et al., successful mHealth development follows six interconnected phases, including development and prototyping, which should be co-led with patients to ensure alignment with their lived experiences.^67^ Once initial MedXR prototypes are developed, it is paramount to pretest them with patients through methods such as surveys, focus groups, prototype testing, storyboards, and advisory team discussions. These tests should be followed by iterative feedback cycles to refine the tools, ensuring they are user-friendly and meet patient needs.^67^ For more details and granular best practices on testing prototypes, refer to Virtual Reality Clinical Outcomes Research Experts (VR-CORE) guidance published by Birckhead et al.^31^ Additional resources on mHealth intervention development also emphasize co-participatory efforts.^68–71^ For instance, Jessen et al. highlight the relevance of gamifying mHealth tools to improve user engagement and adherence, while pointing out the current gap in incorporating user preferences in game design for managing chronic conditions.^72^ Grosjean et al. explore communication models between research teams and patients during mHealth technology development.^73^ Morton et al. apply user-centered design principles to foster collaboration among patients, researchers, and software developers in mHealth creation.^74^ Lastly, Ikwunne et al. emphasize the importance of incorporating socio-cultural contexts during the design stage of mHealth tools.^75^

#### Inclusive testing

To ensure broad accessibility and functionality, MedXR devices should be tested with a diverse patient group, encompassing a range of technology literacy levels, health conditions, and prior experience with immersive technologies. Participant characteristics such as age, socioeconomic status, cognitive function, emotional reactivity, and prior exposure to similar technologies can significantly influence outcomes. To enhance the inclusivity, accessibility, and generalizability of MedXR interventions, it is essential to carefully recruit diverse participant testers, thoroughly document their attributes, and screen for factors like susceptibility to VR-induced motion sickness and the use of common assistive devices such as glasses or hearing aids.^76^

#### Iterative feedback loops

Implement a continuous feedback process where patients review and provide input on each prototype version. This iterative approach allows researchers to make timely adjustments based on patient preferences, ensuring a final product that resonates with end users. For further guidance, see VR-CORE. To maintain consistency among testers and improve the reproducibility of user feedback, researchers should systematically monitor and document environmental distractions that could influence patient feedback during testing, such as background noise, waiting times, calibration delays, or the presence of observers during sessions.^76^

#### Data ownership and IP rights

Establish clear expectations regarding data and intellectual property rights from the outset. Define ownership, access, and patient rights concerning data generated during research, including any potential commercial applications. Collaborative agreements on data sharing or IP co-ownership foster trust and ensure patient contributions are respected, supporting ethical and fair research practices.

### Recruitment and implementation

#### Inclusive recruitment strategies

Partner with patients to develop recruitment strategies that reach a diverse population, ensuring the study sample reflects broader patient demographics. Patient insights help identify effective ways to engage various communities, enhancing recruitment success, as illustrated in the NIH-funded MedXR trial case study.^14^

#### Address participation barriers

Engage patients in identifying and mitigating barriers like time, travel, or technology access. By reducing these obstacles, researchers make the study more accessible, increasing enrollment and retention for a more representative sample.

#### Clear informed consent strategies

Collaborate with patients to create informed consent documents that are accessible, clear, and comprehensive.^17,77^ Patient input ensures that forms use plain language and provide essential information for participants to fully understand the study and its research goals, the potential risks, and their rights, including confidentiality and the voluntary nature of research participation. Research goals can vary depending on the phase of the clinical trial—some studies expose participants to interventions intended to treat or heal with or without standard medical care, while others focus on product improvement. This transparency builds trust, particularly when working with vulnerable populations.^69^

### Data collection and monitoring

#### Identify relevant outcomes

Collaborate with patient partners to pinpoint outcomes that are most meaningful to them, such as patient-reported outcomes. Ensuring that the study’s primary outcome reflects patient priorities enhances the relevance and impact of MedXR research.

#### Safety monitoring

Although there is strong evidence showing the benefits of using VR, some researchers have noted that using VR for long periods can cause “simulator sickness,” otherwise known as cybersickness, which includes symptoms like dizziness, vomiting, and sleepiness.^78^ It is not only important to clearly explain the risks of participating in VR research to study participants but also to involve patient advisors in safety monitoring, including reviewing AEs and consulting them for responses to emerging safety concerns. Patient input provides real-world context to AEs, improving monitoring rigor and fostering trust and transparency with research procedures.

#### Study monitoring

Collaborating with patient partners when designing and implementing study procedures is essential for improving participant retention and engagement. This collaboration can help identify effective incentive strategies and address key motivators of participants for both joining and remaining in MedXR studies.^79,80^ Patients often participate in clinical research out of gratitude toward their health care providers, hope that novel interventions may offer personal benefit, and a desire to contribute to scientific advancement and help future patients.^81^ Other common motivators include study procedures that are simple and non-burdensome (e.g., short, easy-to-complete surveys), as well as the use of intuitive and engaging technology, such as user-friendly software platforms and accessible hardware, especially in the context of MedXR. For example, minimizing technical requirements—such as using VR headsets that are comfortable and require minimal broadband—can reduce barriers to participation. By incorporating patient feedback into the design of these tools and procedures, researchers can create more inclusive, engaging, and user-friendly studies that encourage better compliance and a more inclusive, effective study experience.^82,83^

### Data analysis and dissemination

#### Data interpretation

Include patients in the interpretation of data to contextualize findings in ways meaningful to the patient community. Visualizations should be clear of jargon and accessible to all study participants. Patient advisors can offer essential perspectives on unexpected or contradictory results, suggesting explanations and emphasizing real-world significance for the narrative of the study. Their involvement in crafting sensitive, clear messaging ensures alignment with patient community values and minimizes unnecessary concerns.

#### Transparent data sharing

Develop clear data-sharing processes with patient input, ensuring participants have access to results and understand data usage. Transparency strengthens trust and keeps patients informed about their contributions’ impact.

#### Coauthor publications

Involve patients as coauthors in reports and publications to acknowledge their partnership and ensure findings reflect patient perspectives and priorities.^13,14,30,66^

#### Cross-sector communication

Collaborate with PPI to develop communication strategies that make findings accessible and relevant to patient audiences, enhancing the research’s real-world impact.^77^ Include dissemination strategies that utilize universal design principles, including plain language, engaging videos, and multi-language materials to address all audiences—patients, clinicians, funders, and technologists.

#### Broader community outreach

Work with patients to share findings through advocacy groups, support networks, and social media, in addition to publishing studies in peer-reviewed journals. Patient insights can identify the most effective channels, maximizing the research’s reach and relevance to the broader patient community and general population.

### Post-study feedback

#### Gather patient feedback

After the study, seek patient feedback on the research process to identify strengths and areas for improvement, refining future PPI practices.

#### Strategize feedback use for future studies

Use patient insights to improve the design and conduct of subsequent MedXR studies, fostering continuous enhancement in patient-centered research.

#### Sustain relationships with patient partners

Engage with patient partners post-study to build long-term relationships and integrate PPI into core organizational practices. Creating dedicated roles or feedback loops strengthens ongoing collaboration and aligns research with patient needs.

## Strategies for Success in PPI

Patient engagement is essential for impactful, patient-centered MedXR research, yet it poses unique challenges. Below are key challenges and solutions to navigate them effectively.^47,84,85^

### Educate and support patients with MedXR technology

MedXR technology can be unfamiliar to many, leading to hesitancy.^86–90^ Offer patient-friendly, jargon-free educational materials, interactive demos, and tailored support, helping patients feel informed and confident in their participation.

To embed meaningful PPI in MedXR research, each strategy must align clear goals with actionable steps, address barriers, and include team training. Educate both patients and staff using plain-language resources and empathy-based training. Foster inclusive recruitment, provide technical support, and offer flexible roles to maintain engagement. Empower patients through co-learning, involve them in ethical decision-making, and ensure findings are communicated in an accessible way. These practices enhance equity and effectiveness and ensure the research is grounded in lived experience.

When a patient partner’s availability changes due to personal or medical reasons, teams should respond with flexibility and empathy. Offer various ways to stay engaged, such as shorter tasks, asynchronous input, or pausing participation without pressure. Always acknowledge their contributions and keep the door open for future re-engagement. This flexibility ensures participation remains respectful, sustainable, and patient-centered.

To support technology access and literacy, provide hands-on tutorials, user-friendly guides, and ongoing support.^90–93^ Addressing tech barriers proactively ensures all patients can participate meaningfully, preventing frustration and disengagement.^94^

### Develop inclusive recruitment strategies

Engage a diverse, representative patient group by using culturally sensitive recruitment materials and partnering with advocacy groups.^91–93,95^ Flexible participation options, like virtual meetings, can reduce barriers for underrepresented communities.

### Foster co-learning and equity

Cultivate a research environment where PWLE are held in equal regard to the academic and clinical expertise of researchers and other stakeholders.^10,44–47^ Co-learning means creating opportunities for mutual education—where researchers learn from patients’ lived experiences, and patients gain a clearer understanding of research processes, goals, and constraints. Include structured opportunities for patients to share insights and ensure transparency on how their input shapes research decisions.

### Sustain meaningful roles across research stages

Maintain engagement by offering varied roles for patients, such as iterative prototype testing and data feedback.^14,96,97^ Active involvement and acknowledgment sustain patient interest and commitment. An essential component of successful PPI in medical XR research is the thoughtful organization and ongoing support of patient advocates, advisors, and codesigners. This begins with clearly defining roles and responsibilities, ensuring that participants fully understand what is expected of them and how their contributions will shape the research. Managing expectations—from both researchers and participants—is key to building trust and sustaining engagement. Open and continuous communication should be encouraged, creating a space where feedback is welcomed, and adjustments can be made collaboratively. Additionally, it is important to plan for flexibility: participants may experience changes in health, availability, or interest over time. Establishing protocols for these situations—such as offering alternative ways to contribute or stepping back temporarily without stigma—helps maintain inclusion and respect throughout the project.

### Codesign ethical oversight

Include patients in ethical discussions about data privacy and potential risks. Their input ensures the research aligns with patient rights and values, fostering ethical and respectful study practices.

### Tailor communication for all audiences

Effective communication means making information accessible to everyone—regardless of their background, role, or level of expertise.^15,32,64,65,98–100^ Whether speaking to patients, advocates, researchers, or health care professionals from different specialties, it’s essential to use clear, jargon-free language that bridges knowledge gaps. Visual aids and the use of diverse formats, such as summaries, infographics, videos, and translated materials, may assist with ensuring findings are understandable, meaningful, and actionable for all stakeholders. Tailoring messages to meet a variety of health literacy levels, cultural contexts, and communication preferences also supports meaningful engagement, empowers informed decisions, and promotes interdisciplinary collaboration. By prioritizing clarity and avoiding overly technical or discipline-specific terms, we can create a shared understanding that supports collaboration, informed decision-making, and the equitable use of information across all communities.

## Case Studies

To illustrate the practical application of PPI principles in MedXR research, the following section presents three case studies. These examples highlight different approaches to integrating patient voices throughout the research process, from study design to dissemination of findings. Each case study demonstrates how meaningful collaboration with patient partners can enhance the relevance, effectiveness, and ethical rigor of MedXR interventions. By examining these real-world examples, researchers can gain insights into the challenges and opportunities of patient engagement and explore strategies for successfully implementing these practices in their own work.

### Case study #1: Building trust through inclusive messaging—enhancing racial and ethnic diversity in a MedXR clinical trial

An NIH-funded study evaluating the efficacy of VR for chronic low back pain (cLBP) aimed to enhance diversity in clinical trial participation.^101^ To inform recruitment strategies, focus groups were conducted with non-Hispanic Black (NHB) and Hispanic community members, including participants from the parent trial. Thematic analysis revealed four key barriers to participation: mistrust, lack of interest, cultural differences, and communication gaps.

In response, the study team adapted its recruitment materials and strategies. These changes included physician-endorsed recruitment letters and emails, simplified study documents, and revised advertisement flyers. Notably, the flyer’s messaging was shifted from technical language to patient-centered language, such as changing the title to emphasize “Low Back Pain Relief” rather than clinical terminology.

To ensure cultural and contextual relevance, a subset of focus group participants reviewed the revised materials, offering feedback on language, tone, and alignment with community perspectives. These iterative adaptations led to a significant increase in overall enrollment and improved representation of NHB (*p* = 0.011) and Hispanic (*p* < 0.015) participants.

This case study underscores the value of integrated patient advocates—serving as longitudinal partners across all study phases, from design to recruitment, data interpretation, and dissemination. The broader focus groups provided essential input on recruitment and study design, while the subset enabled deeper collaboration on refining materials and interpreting findings to ensure community-informed research.

### Case study #2: Designing for distance—expanding geographic representation in a MedXR study

In an NIH-funded study comparing the effectiveness of two digital pain management tools—PainTRAINER (a 2D mobile app) and RelieVRx (a 3D VR app)—among rural populations, PPI was integrated across all stages. In the study design stage, a coalition of six academic institutions, including Cedars-Sinai, Duke, and the University of Alabama at Birmingham (UAB), partnered with the American Chronic Pain Association (ACPA) to codesign study strategies and adapt study protocols to the lived experiences of rural patients. In the MedXR prototyping phase, patient partners tested both tools and offered usability feedback that improved onboarding procedures.

Recruitment and consent materials were co-created to ensure clarity and relevance for rural populations, and key participation barriers were identified with potential solutions (i.e., remote participation options during the COVID-19 pandemic). Their insights were crucial in building trust among participants and resulted in the recruitment of 330 patients with chronic pain from rural clinics in California, Louisiana, and Alabama (47%).^102^

Throughout data collection, patient advisors helped define outcomes and select validated surveys that minimized participant burden. During the dissemination phase, preliminary results were shared with patient advisors, who continue to guide efforts to tailor findings for rural populations. In post-study feedback, survey results will be reviewed, and meetings with stakeholders will help identify improvements and establish long-term partnerships. This participatory approach not only ensured a geographically diverse sample but also guaranteed that the research addressed the specific priorities and needs of rural patients.

### Case study #3: Starting with the patient voice—early-stage codesign in MedXR programs using the VR-Core model

Using VR-CORE’s three-phase approach (VR1–VR3) for VR development, several MedXR programs, such as IBS/VR (for gut-directed therapy)^103^ and XAIA (an AI-enabled VR mental health program),^104,105^ prioritized patient engagement from the study design and development, MedXR prototyping, and recruitment phases. Patient feedback during the VR1 content development stage led to 23 modifications in IBS/VR, refining audiovisual elements and content pacing. In XAIA, patient input guided adjustments to AI responses and VR environments, improving therapeutic relevance and immersion. Both programs illustrate how early and iterative patient engagement can shape more effective, user-friendly interventions.

These examples underscore the impact of human-centered design in MedXR research, demonstrating how patient engagement from the outset leads to interventions that are accessible, engaging, and aligned with patient needs.

## Conclusion

PPI is essential in MedXR research to create interventions that are truly patient-centered and impactful. Actively engaging PWLE from the design phase through post-study feedback ensures that MedXR studies remain relevant, ethical, and aligned with real-world patient needs.

This AMXRA expert consensus offers a framework for building strong patient partnerships, drawing on successful strategies from organizations like PCORI and NIH. It provides researchers, developers, and clinicians with a practical approach to engaging patients in MedXR studies, leading to more meaningful outcomes, higher patient satisfaction, and the development of widely adopted interventions. We acknowledge that financial and resource constraints can hinder some teams from fully implementing this expert consensus, especially those without direct patient access from health systems or academic institutions. While this limitation is beyond the scope of this document, we encourage collaborations between large and small institutions and recommend that both federal and nonfederal funding sources allocate special funds to support diverse PPI efforts. Leveraging community partnerships and applying the PPI principles in this guide can help overcome these challenges.

When patients are treated as equal partners, research outcomes are better aligned with their needs and priorities. As MedXR evolves, continuous PPI will be crucial in shaping research that truly serves the people it aims to benefit. Collaborating with patient advocates will also be key in influencing policies that support patient-centered MedXR technologies. Patient insights can shape regulations, funding priorities, and policies focused on accessibility, data protection, and equitable access.

As MedXR progresses, the research community must refine best practices, adapting to the unique challenges and opportunities presented by this technology. Most MedXR research to date has been consultative, as shown in Fig. 3. We envision the AMXRA expert consensus will provide a framework for teams to adopt a more collaborative and integrated leadership style in PPI for MedXR trials. This ongoing commitment will ensure that MedXR interventions evolve in ways that remain closely aligned with the values and needs of the patient communities they aim to serve. Finally, co-leadership, trust, and transparency must be prioritized, with institutions supporting researchers in fostering lasting PPI. As MedXR research continues to grow, PPI must remain at its core.

## Statement on Use of Artificial Intelligence

AI was employed in the preparation of this document to assist with editing and refining the text. The original content was written by the authors, and AI was utilized to enhance clarity and coherence. The final document reflects the authors’ insights and expertise, supported by AI-driven editorial tools.

**NB:** In this expert consensus, “patient” is used inclusively to mean anyone with lived experience of a health condition, disability, or interaction with the health care system—regardless of diagnosis or treatment status. This includes people with chronic or acute conditions, those at risk or in recovery, individuals from underserved communities, and caregivers or family members who provide unpaid support.

PPI means actively partnering with PWLE, caregivers, and the public in health research and care planning. Rather than being passive subjects, patients, who are “experts by experience,” bring essential insights that strengthen the relevance, ethics, and impact of research, and help shape priorities, study design, materials, and the sharing of results. PPI ensures research is relevant, equitable, and grounded in real-world needs, increasing trust, transparency, and the impact of outcomes. In MedXR, their involvement is not optional—it is essential to creating equitable, effective, and human-centered technologies and providing insights that enhance the relevance, ethics, and impact of research.

## Abbreviations Used

AMXRA: American Medical Extended Reality Association
CDMRP: Congressionally Directed Medical Research Programs
MedXR: Medical extended reality
NIH: National Institutes of Health
PCORI: Patient Centered Outcomes Research Institute
PPI: Patient and public involvement
PWLE: Patients with lived experiences

## Glossary

**Patient and Public Involvement (PPI)** refers to the active partnership of patients, caregivers, and members of the public in shaping health research and health care planning. Unlike traditional models where patients are passive subjects or care recipients, PPI emphasizes collaboration—working *with* or *by* patients rather than *to*, *about*, or *for* them. This involvement can take many forms, including codeveloping research questions, advising on study design, reviewing consent forms, interpreting findings, and helping to share results in accessible ways. The goal is to ensure that research and health care decisions are relevant, equitable, and aligned with the lived experiences of the people they are intended to serve. By integrating diverse voices, PPI increases transparency, trust, and the likelihood that outcomes will be meaningful and adopted in real-world settings.^26^

**Person(s) with Lived Experience (PWLE)** are regarded as “*experts by experience*” in the scope of their firsthand experience with a diagnosis or health condition.^106^

**Medical Extended Reality (MedXR)** involves the intersection of health care and immersive technologies such as virtual reality (VR), augmented reality (AR), and mixed reality (MR) that extend or enhance the medical experience.^1^

**Virtual Reality** is a virtual-world immersive experience that may require a headset to completely replace a user’s surrounding view with a simulated, immersive, and interactive virtual environment.^107^

**Augmented Reality** is a real-world augmented experience with overlaying or mixing simulated digital imagery with the real world as seen through a camera or display, such as a smartphone or head-mounted or heads-up display. Digital imagery may be able to interact with real surroundings (often controlled by users). This is sometimes referred to as mixed or merged reality.^107^

**Patient-Centered Outcomes Research Institute (PCORI)** is an independent, nonprofit research funding organization that seeks to empower patients and others with actionable information about their health and health care choices.^28^

**National Institutes of Health (NIH)**, part of the U.S. Department of Health and Human Services, is the nation’s medical research agency—making important discoveries that improve health and save lives.^108^

**Congressionally Directed Medical Research Program (CDMRP)** originated in 1992 via a Congressional appropriation to foster novel approaches to biomedical research in response to the expressed needs of its stakeholders—the American public, the military, and Congress.^109^
